# Predictive ability of Child Psychosis-Risk Screening System exceeds its immediate diagnostic ability

**DOI:** 10.1192/j.eurpsy.2026.11153

**Published:** 2026-07-31

**Authors:** Y. Hamasaki, Y. Sakaue, S. Michikoshi, T. Nakayama, S. Ueba, Y. Mutsuda, M. Isobe, T. Hikida

**Affiliations:** 1 Faculty of Contemporary Society, Kyoto Women’s University, Kyoto; 2 Department of Pediatrics, Shiga University of Medical Science, Shiga; 3 Faculty of Data Science, Kyoto Women’s University, Kyoto; 4 Department of Pediatrics, Saiseikai Moriyama Municipal Hospital, Shiga; 5 Department of Psychiatry, Kyoto University Graduate School of Medicine, Kyoto; 6 Laboratory for Advanced Brain Functions, Osaka University, Osaka, Japan

## Abstract

**Introduction:**

Patients who experience their first psychotic episode often have a long history of pediatric treatment for psychosomatic symptoms. However, it is difficult to objectively assess the risk of developing psychosis in pediatric clinical practice.

**Objectives:**

The aim of this prospective study was to evaluate the predictive ability of the Child Psychosis Risk Screening System (CPSS), which was designed to evaluate the development of schizophrenia spectrum disorder (SSD) by observing the outcomes of pediatric and psychiatric outpatients.

**Methods:**

Overall, 491 outpatients aged 6–18 years who visited the pediatric and psychiatric departments of university and community hospitals were enrolled. Patients were assessed based on the Child Behavior Checklist (CBCL) and clinical data (sex, age, month of birth, chief complaint, diagnosis, and any signs of abuse, bullying, or withdrawal). Using the CBCL data, the CPSS calculates the risk of developing SSD. The presence or absence of SSD was confirmed 1–4 years later. Receiver operating characteristic (ROC) analysis was used to verify both the immediate diagnostic and predictive abilities of the CPSS. The Light Gradient-Boosting Machine (LightGBM) algorithm was used to calculate the variable importance of each clinical data point in predicting the onset of SSD.

**Results:**

The ROC analysis showed that the CPSS adequately predicted the onset of SSD (area under the curve = 0.906, 95% confidence interval [CI]: 0.872–0.940), and its predictive ability exceeded its immediate diagnostic ability (area under the curve = 0.841, 95% CI: 0.781–0.900). The LightGBM results revealed the importance of the CPSS in predicting SSD onset was higher than that of any other variable, including the Thought Problems subscale of the CBCL.

**Conclusions:**

The CPSS shows adequate predictive power for the development of SSD and may be used as an objective adjunctive diagnostic method to screen children at risk for SSD who require early referral to psychiatry for psychosomatic disorders.

**Disclosure of Interest:**

None Declared

